# Distinct vaginal microbiome and metabolome profiles in women with preterm delivery following cervical cerclage

**DOI:** 10.3389/fcimb.2025.1444028

**Published:** 2025-02-11

**Authors:** Jun Zhang, Li Li, Mengjun Zhang, Jiaoning Fang, Zhimin Xu, Yijing Zheng, Zhi Lin, Mian Pan

**Affiliations:** Department of Obstetrics and Gynecology, Fujian Maternity and Child Health Hospital, College of Clinical Medicine for Obstetrics and Gynecology and Pediatrics, Fujian Medical University, Fuzhou, Fujian, China

**Keywords:** preterm birth, complete blood cell indices, vaginal microbiota, vaginal metabolites, metabolic pathway

## Abstract

Preterm birth (PTB) is a major cause of infant morbidity and mortality. The aim of this study was to investigate the effect of vaginal microbiota and metabolites on the outcome of pregnant women. In this study, a total of 127 pregnant women provided written informed consent prior to enrollment in accordance with the approved institutional guidelines, but only 45 pregnancies met the experimental requirements, and then blood and cervical vaginal fluid (CVF) samples were collected before delivery (at the second week after cervical cerclage). Pregnant women with PTB exhibited high white blood cell and neutrophil contents, high neutrophil-to-lymphocyte ratio (NLR), and high systemic inflammation response index (SIRI) in the blood. Vaginal microbiome revealed that the proportion of beneficial bacteria (including *Lactobacillus*, [*Ruminococcus*] *gnavus group*, and *Megamonas*) significantly decreased in the PTB group, and the proportion of harmful bacteria (including *Desulfovibrionaceae*, *Helicobacter*, and *Gardnerella*) significantly increased, which is strongly related to the biochemical parameters of blood (white blood cells, neutrophils, NLR, and SIRI). In addition, vaginal metabolomics-based liquid chromatography–Orbitrap–tandem mass spectrometry (LC-Orbitrap-MS/MS) found that the alteration in vaginal metabolites in pregnant women with PTB is involved in starch and sucrose metabolism; arginine and praline metabolism; galactose metabolism; purine metabolism; arginine metabolism; tryptophan metabolism and N-glycan biosynthesis; cysteine and methionine metabolism; taurine and hypotaurine metabolism; amino acid metabolism; propanoate metabolism; valine, leucine, and isoleucine biosynthesis; glycine, serine, and threonine metabolism; and steroid hormone biosynthesis. These results elaborated that distinct vaginal microbiome and metabolome profiles in women with preterm delivery following cervical cerclage provide valuable information for establishing the prediction models for PTB.

## 1 Introduction

Preterm birth (PTB), defined as birth prior to the completion of 37 weeks of gestation, is the world’s leading cause of neonatal and childhood death. According to a previous investigation, approximately 152 million babies are born every year, including spontaneous PTB and spontaneous preterm labor with ruptured membranes (Preston et al., 2024). Among premature infants, approximately 45% are diagnosed as spontaneous preterm labor with intact membranes, and approximately 30% are diagnosed as spontaneous preterm labor with ruptured membranes (Beernink et al., 2023). Compared with term infants, the main organ systems of preterm infants have incomplete development and are highly vulnerable to the external environment, which elevates the risk of multiple complications, such as respiratory illnesses, cerebral palsy, infections, and blindness (Liu et al., 2012). At present, some strategies are widely used in clinical practice to reduce the occurrence of PTB and the risk of multiple complications. Among them, cervical cerclage is widely used in the preventive treatment of PTB and preterm premature rupture of membranes by supporting the tissue, preserving the cervical mucus plug, and preventing ascending vaginal infection (Cassardo et al., 2024; Seyama et al., 2022). As an invasive procedure, a cervical cerclage can potentially elevate the risk of infection and dysbiosis of the vaginal microbiome. While some women with successful outcomes delivered at full term, others may deliver prematurely following the procedure. This discrepancy of cerclage outcome is further associated with dysbiosis of the vaginal microbiome and even subclinical infections (Bauer et al., 2020; Bayar et al., 2020). The identification of “hidden” and key infectious factors is a crucial step in determining which patient populations may derive benefit from cervical cerclage.

Many microorganisms reside in the human vagina, mainly bacteria, forming a complex ecosystem known as the vaginal microbiota (Petricevic et al., 2014). Among these, *Lactobacillus* is one of the major bacteria in the vagina, and plays the most important role in improving vaginal homeostasis. It is reported that the proportion of vaginal *Lactobacillus* was significantly reduced in women with vaginal diseases, such as bacterial vaginitis, trichomoniasis, and vulvovaginal candidiasis (Zeng et al., 2023). In contrast, a high abundance of *Lactobacillus* in the vagina is beneficial for suppressing the growth of harmful bacteria and elevating the content of short-chain fatty acids (Hong et al., 2021). Short-chain fatty acids are the most important metabolites and are critical factors in suppressing the development of vaginal diseases. In addition, some studies have also shown that an increased abundance of *Lactobacillus* effectively suppresses the secretion of proinflammatory cytokines, alleviates oxidative stress, and regulates the composition of the vaginal microbiota (Guo et al., 2020). However, the reduction of *Lactobacillus* species elevates the risk of vaginal diseases, which may be associated with the high rate of PTB (Gershuni et al., 2021). Another study also found that a high abundance of *Gardnerella* promotes the morbidity and development of vaginal diseases by utilizing amines to produce amino acids (Noue et al., 2024). Noteworthily, changes in the vaginal microbiota induce vaginal metabolites that have a significant and varied impact on overall health and are detectable in a range of biological tissues, including the colon, liver, brain, and vagina. However, the effect of vaginal metabolites on PTB is still unclear.

In the present study, 127 participants were recruited from the Fujian Maternity and Child Health Hospital (Fuzhou, China) from July 2021 to December 2023, but only 45 participants met the experimental requirements (Fettweis et al., 2019). The aim was to comprehensively explore the vagina microbiota and metabolites of pregnant women with PTB and term birth at the 14th day after cervical cerclage using high-throughput sequencing and untargeted metabolomics-based liquid chromatography–Orbitrap–tandem mass spectrometry (LC-Orbitrap-MS/MS), respectively. Key microbial phylotypes and differential metabolic features in the vagina of pregnant women with PTB after cervical cerclage were screened using statistical analysis, which provides useful information for establishing the prediction models to identify pregnancies at risk for PTB and for developing new therapy for PTB.

## 2 Materials and methods

### 2.1 Participant recruitment and sample collection

**Figure 1:**
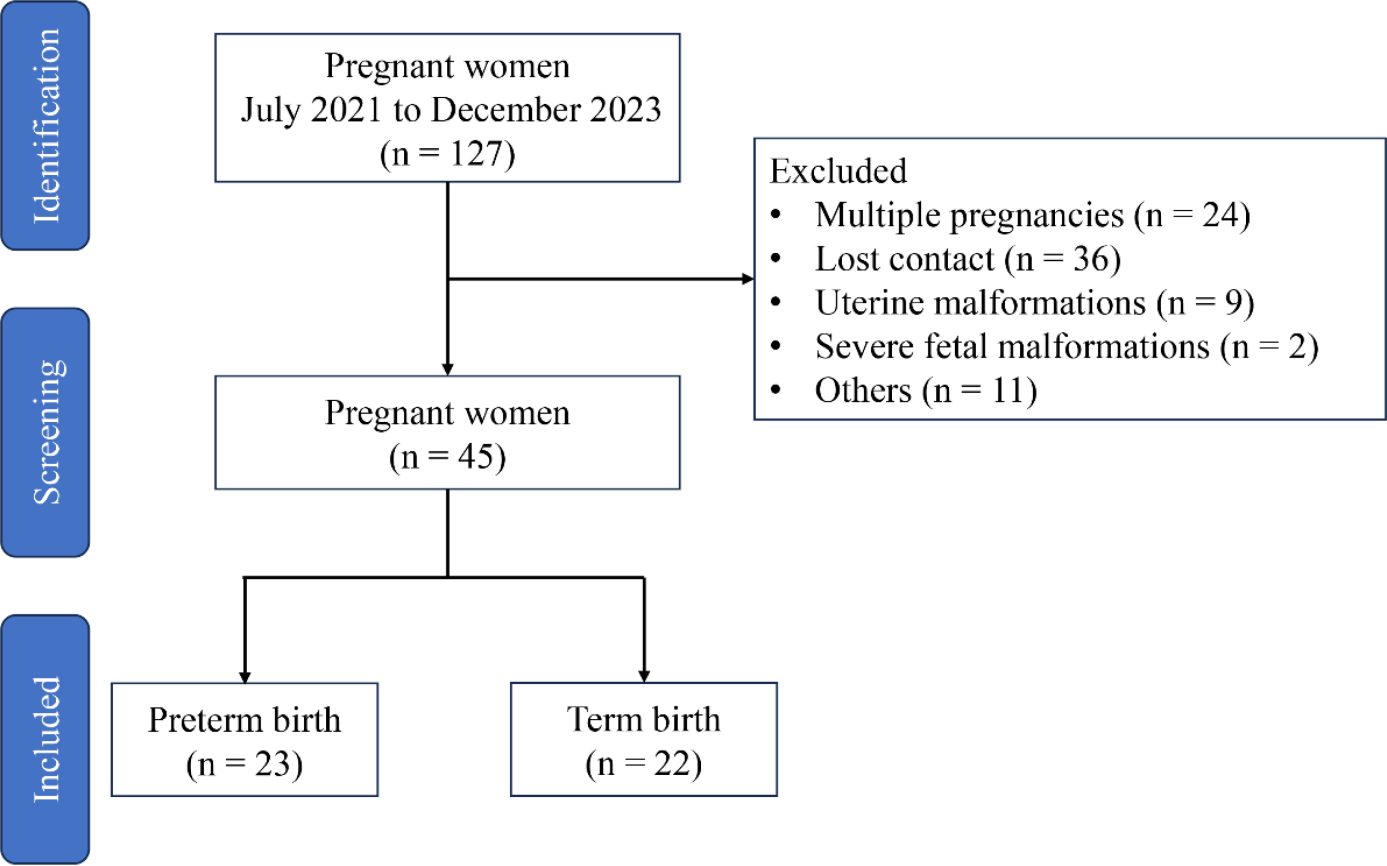
Study flow diagram.

Participants were recruited from the Fujian Maternity and Child Health Hospital (Fuzhou, China) from July 2021 to December 2023, and the trial was approved by the Ethics Committee of Fujian Maternity and Child Health Hospital (Approval No. 2021KLR601). A total of 127 participants offered written informed consent before enrollment in accordance with the approved institutional guidelines, but only 45 participants met the experimental requirements (excluding 24 cases of multiple pregnancies, 36 cases of lost contact, 9 cases of uterine malformations, 2 cases of severe fetal malformations, and 11 cases of maternal or fetal indications for iatrogenic preterm delivery) (**Figure 1**). The recruitment criteria are as follows: (1) 21 and 35 years old; (2) BMI from 18 to 28; (3) gestational age from 18 to 24 weeks; (4) singleton pregnancy; (5) cervical dilation of ≥1 cm; (6) complete membrane; and (7) no vaginal bleeding. Patients with certain conditions have been excluded: (1) labor onset; (2) membrane injury; (3) clinical chorioamnionitis, manifested as temperature of more than 38.0°C; heart rate of more than 100 beats/min; fetal heart rate of more than 160 beats/min; uterine tenderness; malodorous vaginal discharge; abnormal peripheral blood leukocyte count [white blood cell (WBC) count of more than 15 × 10^9^/L or left shift in neutrophils]; (4) placental abruption; (5) fetal congenital anomalies or maternal medical or surgical complications requiring termination of pregnancy; and (6) twin or multiple pregnancies.

The blood and cervical vaginal fluid (CVF) samples (from the posterior vaginal fornix) were collected once on the 14th day after cervical cerclage (McDonald Swab), transferred to 0.75 mL of phosphate-buffered saline (containing protease inhibitors and EDTA), and stored at −80°C until used.

### 2.2 Data collection

The historical outcomes of pregnant participants were collected and recorded, including maternal age, body mass index (BMI), abortion, and fertility frequency. All participants were divided into two groups according to the outcomes of the pregnancies: the post-cerclage PTB (*n* = 23) group and the post-cerclage term birth (TB, *n* = 22) group.

### 2.3 Complete blood cell index analysis

The blood was collected after the cervical cerclage procedure for 2 weeks, and the WBC, neutrophil, lymphocyte, monocyte, platelet, and C-reactive protein (CRP) levels were detected using flow cytometry (XE-3000, SYSMES, Kobe, Japan). Meanwhile, neutrophil-to-lymphocyte ratio (NLR), platelet-to-lymphocyte ratio (PLR), systemic immune inflammation index (SII), and systemic inflammation response index (SIRI) were calculated according to a previous report (Sahin et al., 2023).

### 2.4 High-throughput sequencing

The sequencing analysis of vaginal microbiota was implemented by the MiSeq platform according to a previous study with minor modifications (Guo et al., 2023a). In brief, total bacterial DNA from the CVF sample was extracted using a commercial kit (MoBio, Carlsbad, CA, USA), and then the V3–V4 regions of bacterial 16S rRNA genes were amplified using broad-range bacterial primers, namely, 338F primers (5′-CCTAYGGGRBGCASCAG-3′) and 806R primers (5′-GGACTACHVGGGTWTCTAAT-3′). PCR products were further purified using 2.0% agarose gel electrophoresis, the target fragment was collected using the Agencourt AMPure XP Kit (Hangzhou, China), and then the content of each sample was measured by a Nanodrop 2000 spectrophotometer (Thermo Fisher Scientific, CA, USA). Sequencing libraries consisted of equal concentrations of each sample, and their quality was assessed by a Nanodrop 2000 spectrophotometer and was then analyzed on an Illumina NovaSeq 6000 platform at Shanghai Biotree Biotech. Co., Ltd. (Shanghai, China).

The raw data were filtered, denoised, and merged; chimera was removed using Microbial Ecology software (v 2.0); and the high-quality sequences were collected and grouped into operational taxonomic units (OTUs) with similarities of more than 97%. Taxonomy annotation analysis was carried out on the OTU sequences by the Mothur approach and the SSU rRNA database of SILVA138.1. Before conducting alpha-diversity analysis, a sampling depth of 48,000 was randomly selected to balance the differences in sequencing depth. Alpha-diversity of vaginal microbiota was analyzed according to ASV. Principal coordinates analysis (PCoA) based on PERMANOVA (Jaccard distance) and robust principal component analysis (RPCA) were used to assess the overall differences of vaginal microbiota between the PTB and TB groups, and the key microbial phylotypes (at the genus level) were screened based on Welch’s *t*-test by the STAMP software (v 1.8.2). The raw data from high-throughput sequencing that support the findings of this study are openly available at http://www.ncbi.nlm.nih.gov/bioproject/1122359 (Reference number: PRJNA1122359).

### 2.5 Untargeted metabolomics analysis

Untargeted metabolomics analysis of CFV was carried out by LC-Orbitrap-MS/MS according to a previous report with minor modifications (Guo et al., 2023b). In brief, the vaginal contents were freeze-dried, weighted, and extracted using the organic solution (methanol: acetonitrile = 1:1). The mixture solution was sufficiently vibrated using a high-throughput oscillator, and placed at a 0–4°C environment. After 2 h of stillness, the supernatant of each sample was collected by centrifugation (14,000 rpm, 10 min, 4°C) and then dried at 25°C under a vacuum environment. The sediment of each sample was resuspended using the organic solution (methanol: acetonitrile = 1:1), and the supernatant was collected by centrifugation (14,000 rpm, 10 min, 4°C) and then filtrated through a 0.22-μm aqueous membrane. Quality control samples consisted of an equal volume of each sample to assess the stability of instruments during the experiment.

The vagina metabolic profiling was analyzed using LC-Orbitrap-MS/MS with an ACQUITY UPLC BEH Amide (50 × 2.1 mm, 1.7 µm; Waters, Milford, USA). Among these, MS detection of metabolites was carried out on an Orbitrap Exploris 120 (Thermo Fisher Scientific, USA) with an ESI ion source in both the positive and negative modes, the mobile phase A containing 0.1% formic acid and 5 mM ammonium acetate, and the mobile phase B was acetonitrile. The raw data were preliminarily treated using the ProteoWizard software and R software (v 4.2.1), including peak alignment, peak identification, and deconvolution. The metabolites were identified by accuracy mass (<5 ppm) and MS/MS data, which were matched with the metabolic public databases (massbank, mzcloud, HMDB, and KEGG) (at the level 2) (Zhao et al., 2024). Principal components analysis (PCA, based on PERMANOVA-Euclidean distance), partial least squares discriminant analysis (PLS-DA, cross-validation: fivefold), sparse partial least squares discriminant analysis (SPLS-DA, cross-validation: fivefold), and orthogonal partial least squares discrimination analysis (OPLS-DA, Set permutation numbers: 20) of vaginal metabolomics were carried out using MetaboAnalyst 6.0 (https://www.metaboanalyst.ca/). The differential metabolic features [VIP > 1.0, *p* < 0.05, and fold change (FC) threshold: 2] between the TB and PTB groups was processed by log transformation (base 10) and auto scaling (mean-centered and divided by the standard deviation of each variable) and then visualized by R software (v 4.4.2). The raw data from untargeted metabolomics based on LC-Orbitrap-MS/MS that support the findings of the present study are openly available at www.ebi.ac.uk/metabolights/MTBLS11563 (Reference number: MTBLS11563).

### 2.6 Statistical analysis

All data were presented as mean value ± standard deviation. One-way ANOVA and Student’s *t*-tests were used to examine differences between groups. The Shapiro–Wilk test was applied to assess the normality of data before conducting Student’s *t*-tests. For data that did not follow a normal distribution, the Mann–Whitney rank-sum test was used to compare two groups. *t*-tests and Wilcoxon rank-sum tests with FDR correction for multiple comparisons were conducted. Significant differences were identified at *p* < 0.05.

## 3 Results

### 3.1 Cohort characteristics

The general characteristics of the participants in this study are listed in **Table 1**. The clinical characteristics of the participants exhibit gestational history data in which 51.11% (*n* = 23) of the participants were term birth and 48.89% (*n* = 22) were PTB. There was no significant difference in maternal age and BMI between the TB and PTB groups (*p* > 0.05), namely, the mean maternal age in the PTB group was 31.09 ± 3.93 years with a BMI of 23.54 ± 2.33 and the mean maternal age in the TB group was 30.32 ± 3.77 years with a BMI of 23.19 ± 3.68. In contrast, the body weight of newborns in the PTB group (1,939.76 ± 587.81 g) was significantly lower than that in the TB group (3,158.86 ± 456.62) (*p* < 0.05).

**Table 1:** Descriptive statistics of study participants.

Characteristic	PTB (*n* = 22)	TB (*n* = 23)	*p*
Maternal age (years)	31.09 ± 3.93	30.32 ± 3.77	0.51
BMI (kg/m^2^)	23.54 ± 2.33	23.19 ± 3.68	0.70
Gravida	2.65 ± 1.34	2.50 ± 1.01	0.58
Parity	0.57 ± 0.66	0.55 ± 0.60	0.67
Cervical length (cm)	1.28 ± 1.53	1.86 ± 1.45	0.2
Gestational age at delivery (weeks)	32.25 ± 4.99	38.50 ± 1.00	9.5×10^−9^
Birth weight (g)	1939.76 ± 587.81	3158.86 ± 456.62	4.4×10^−9^
White blood cells (10^9^/L)	11.37 ± 1.82	9.37 ± 1.97	0.001
Neutrophil (10^9^/L)	8.75 ± 1.74	6.88 ± 1.54	0.0004
Lymphocyte (10^9^/L)	1.85 ± 0.51	1.82 ± 0.58	0.87
Monocyte (10^9^/L)	0.61 ± 0.16	0.55 ± 0.17	0.19
Platelet (10^9^/L)	220.91 ± 61.08	219.36 ± 52.65	0.93
NLR	5.22 ± 2.22	4.05 ± 1.29	0.036
PLR	127.70 ± 46.11	127.09 ± 33.11	0.96
SII	742.30 ± 620.67	536.52 ± 290.37	0.16
SIRI	3.24 ± 1.74	2.16 ± 0.78	0.011
CRP	20.27 ± 17.25	12.63 ± 13.57	0.11

The complete blood cell indices of pregnant women were measured, including WBCs, neutrophils, lymphocytes, monocytes, and platelets. Compared with the TB group, the levels of blood WBCs and neutrophils significantly increased in the PTB group (*p* < 0.01). However, there were no significant differences in the serum levels of lymphocytes, monocytes, platelets, and CRPs between the TB and PTB groups (*p* > 0.05). In addition, the blood NLR and SIRI levels were significantly elevated in the PTB group compared with the TB group (*p* < 0.05), while the PLR and SII levels in the blood were slightly increased (*p* > 0.05). This result revealed that complete blood cell indices are strongly related to the risk of PTB.

### 3.2 Alteration of vaginal microbiota in pregnant women with PTB after cervical cerclage

**Figure 2:**
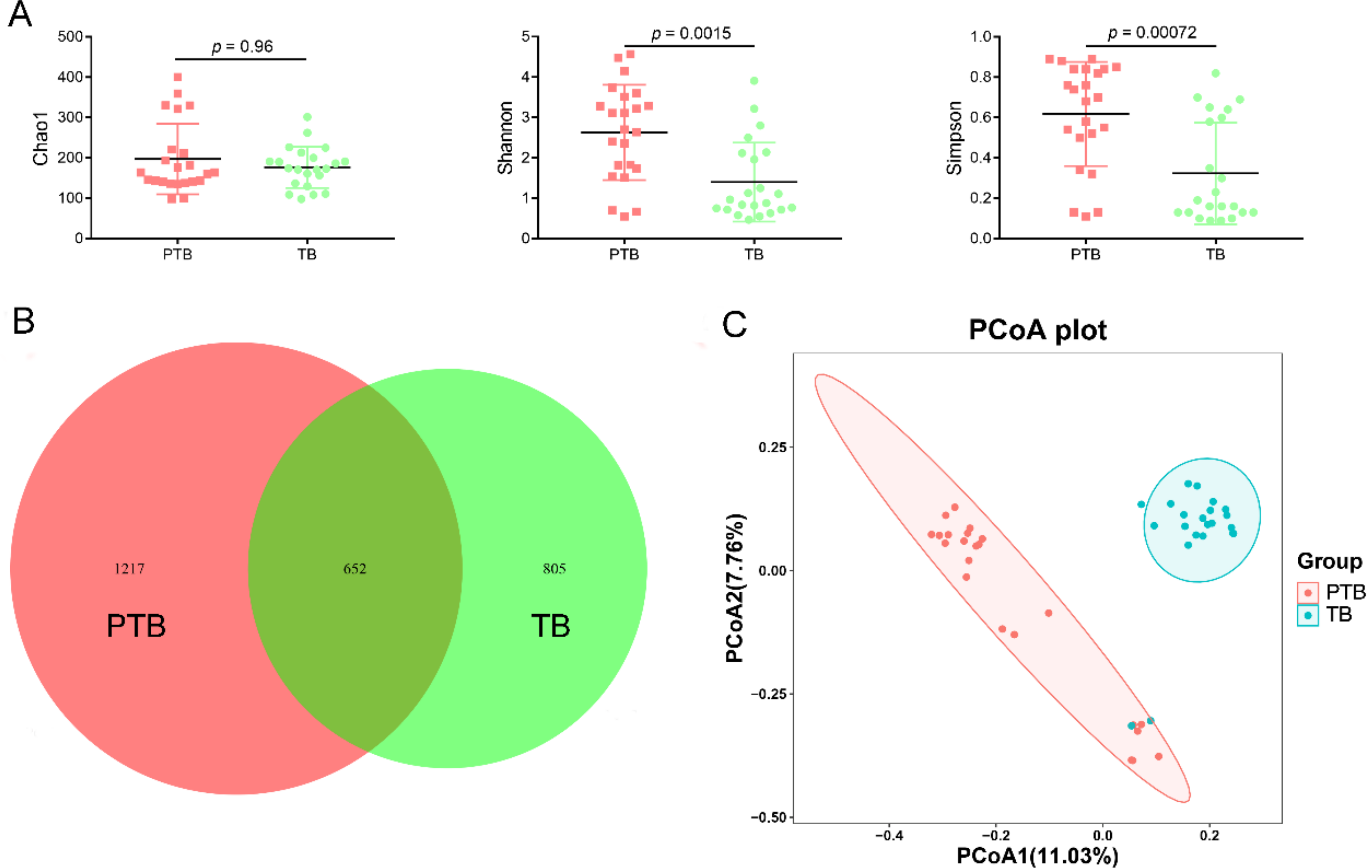
The diversity of between pregnant women with preterm birth and term birth after cervical cerclage. **(A)** Alpha-diversity (Chao1, Shannon, and Simpson indices). **(B)** Venn diagram for the two groups. **(C)** PCoA. PCoA was expressed using the Jaccard distance.

Vaginal microbiota plays the most important role in birth outcomes; thus, the diversity and composition of vaginal microbiota between the TB and PTB groups were explored. There was no significant difference in the Chao1 index between TB and PTB groups (*p* > 0.05), but the Shannon and Simpson indices were significantly elevated in the PTB group compared with the TB group (*p* < 0.05) (**Figure 2A**). The result of the Venn diagram suggested that the PTB group had 1,217 unique features, but the TB group had only 805 unique features (**Figure 2B**). Furthermore, PCoA and RPCA were extensively applied to assess the overall changes of the vaginal microbiota among the difference groups. The results of PCoA and RPCA displayed a clear separation of the vaginal microbiota between the TB and PTB groups, suggesting that vaginal microbiota composition is associated with PTB (**Figure 2C** and **Supplementary Figure S1**).

### 3.3 Screening for key microbial phylotypes

**Figure 3:**
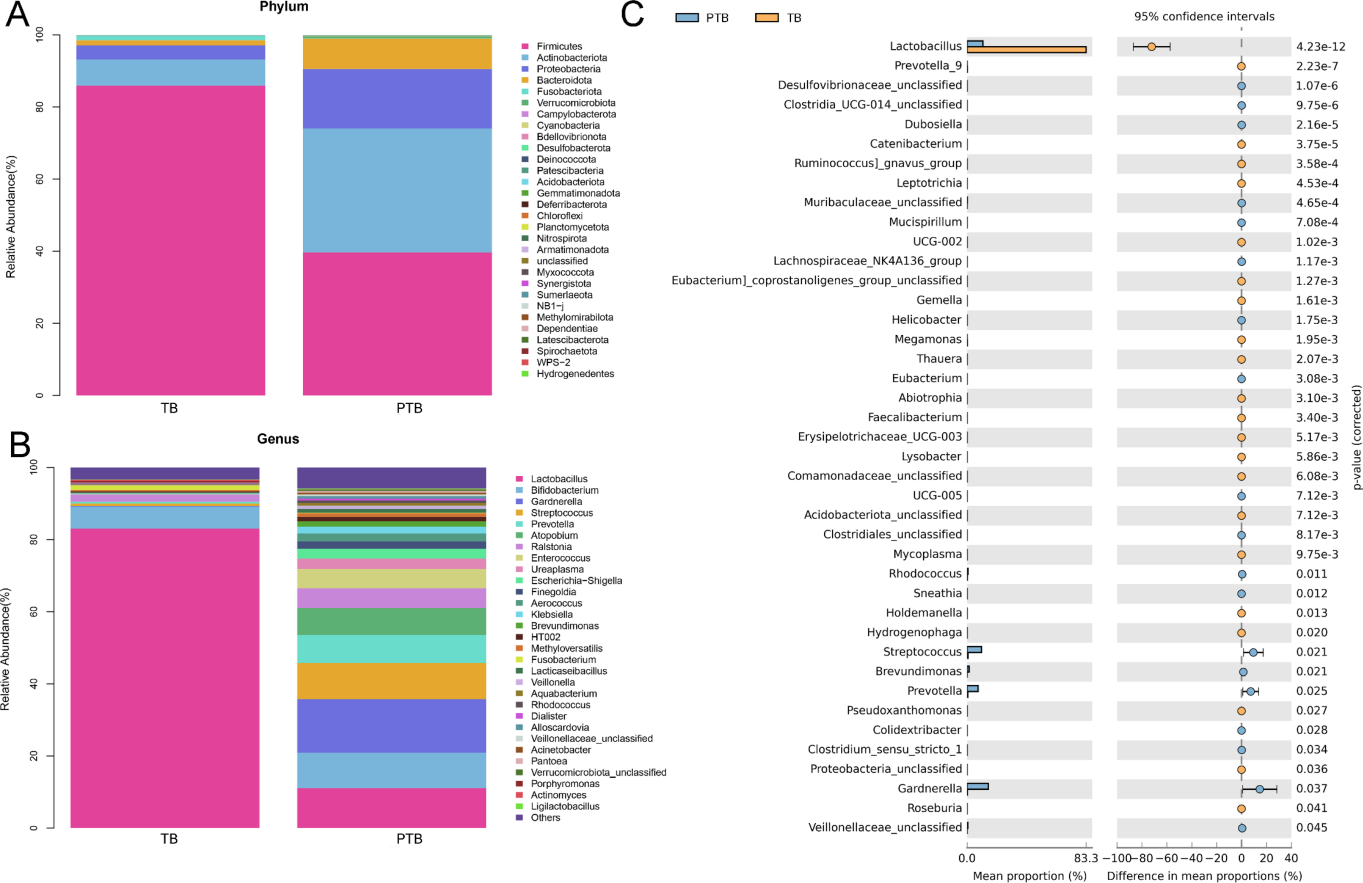
The alteration in vaginal microbiota between the TB and PTB group. **(A)** The proportion of vaginal microbiota at the phylum level (top 30). **(B)** The proportion of vaginal microbiota at the phylum level (top 30). **(C)** Extended error bar analysis of vaginal microbiota was expressed using Welch’s *t*-test.

At the phylum, a total of 30 phyla were detected in both the PTB and TB groups in this study (**Figure 3A**). Among them, Firmicutes, Actinobacteriota, Proteobacteria, Bacteroidota, and Fusobacteriota were the major bacteria in both the TB and PTB groups, accounting for more than 90%. The proportion of Firmicutes was reduced in the PTB group compared with the TB group, whereas the proportions of Actinobacteriota, Proteobacteria, Bacteroidota, and Fusobacteriota were increased. At the genus level, *Lactobacillus* was the major bacterium in the TB group, accounting for more than 90% (**Figure 3B**). However, the most abundant taxa identified in the CVF samples from the PTB group were *Lactobacillus*, *Bifidobacterium*, *Gardnerella*, *Streptococcus*, *Prevotella*, *Ralstonia*, *Enterococcus*, *Ureaplasma*, and *Escherichia*-*Shigella*, accounting for more than 90%. As shown in **Figure 3C**, the relative abundance of *Lactobacillus*, *Prevotella*_*9*, *Catenibacterium*, *Ruminococcus*_*gnavus*_*group*, *Leptotrichia*, *UCG*-*002*, *Eubacterium*_*corprostanoligenes*_*group*_*unclassified*, *Gemella*, *Megamonas*, *Thauera*, *Abiotrophia*, *Faecalibacterium*, *Erysipelotrichaceae*_*UCG*-*003*, *Lysobacter*, *Comamonadaceae*_*unclassified*, *Acidobacteriota*_*unclassified*, *Mycoplasma*, *Holdemanella*, *Hydrogenophaga*, *Pseudoxanthomonas*, *Proteobacteria*_*unclassified*, and *Roseburia* were decreased in the PTB group compared with the TB group, while the relative abundance of *Desulfovibrionaceae*_*unclassified*, *Clostridia*_*UCG*-*014*_*unclassified*, *Muribaculaceae*_*unclassified*, *Lachnospiraceae*_*NK4A136*_*group*, *Helicobacter*, *Eubacterium*, *UCG-005*, *Clostridiales*_*unclassified*, *Rhodococcus*, *Sneathia*, *Streptococcus*, *Brevundimonas*, *Prevotella*, *Colidextribacter*, *Clostridium*_*sensu*_*stricto*_1, *Gardnerella*, and *Veillonellaceae*_*unclassified* were increased.

### 3.4 The association between the complete blood cell indices and key microbial phylotypes

**Figure 4:**
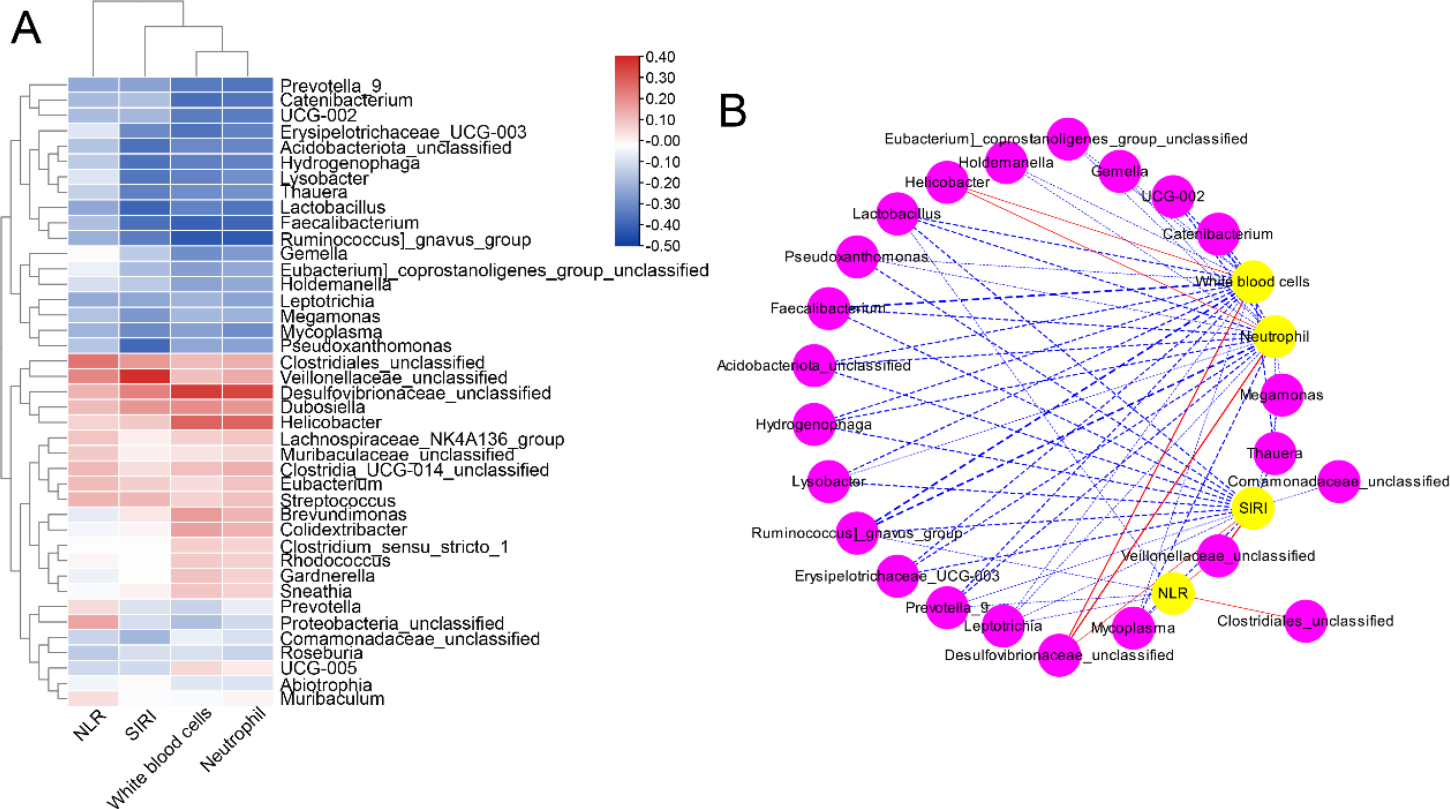
Heatmap **(A)** and network **(B)** analysis of Spearman’s correlation between the complete blood cell indices and the differential abundance of vaginal microbiota (at genus levels).

The correlation between the essential complete blood cell indices and the differential abundance of vaginal microbiota (at the genus level) was analyzed by Spearman correlation analysis (**Figures 4A, B**). *Prevotella 9*, *Catenibacterium*, *UCG*-*002*, *Erysipelotrichaceae*_*UCG*-*003*, *Acidobacteriota*_*unclassified*, *Hydrogenophaga*, *Lysobacter*, *Thauera*, *Lactobacillus*, *Faecalibacterium*, *Ruminococcus gnavus*_*group*, *Gemella*, *Eubacterium*_*coprostanoligenes*_*group*_*unclassified*, *Holdemanella*, *Leptotrichia*, *Megamonas*, *Mycoplasma*, and *Pseudoxanthomonas* were negatively associated with neutrophils, WBCs, NLR, and SIRI. In addition, *Clostridiales*_*unclassified*, *Veillonellaceae*_*unclassified*, *Desulfovibrionaceae*_*unclassified*, *Dubosiella*, *Helicobacter*, *Lachnospiraceae*_*NK4A136*_*group*, *Muribaculaceae*_*unclassified*, *Clostridia*_*UCG*-*014*_*unclassified*, *Eubacterium*, *Streptococcus*, and *Brevundimona* were positively associated with neutrophils, WBCs, NLR, and SIRI.

### 3.5 Alteration of vagina metabolic profiling in pregnant women with PTB after cervical cerclage

**Figure 5:**
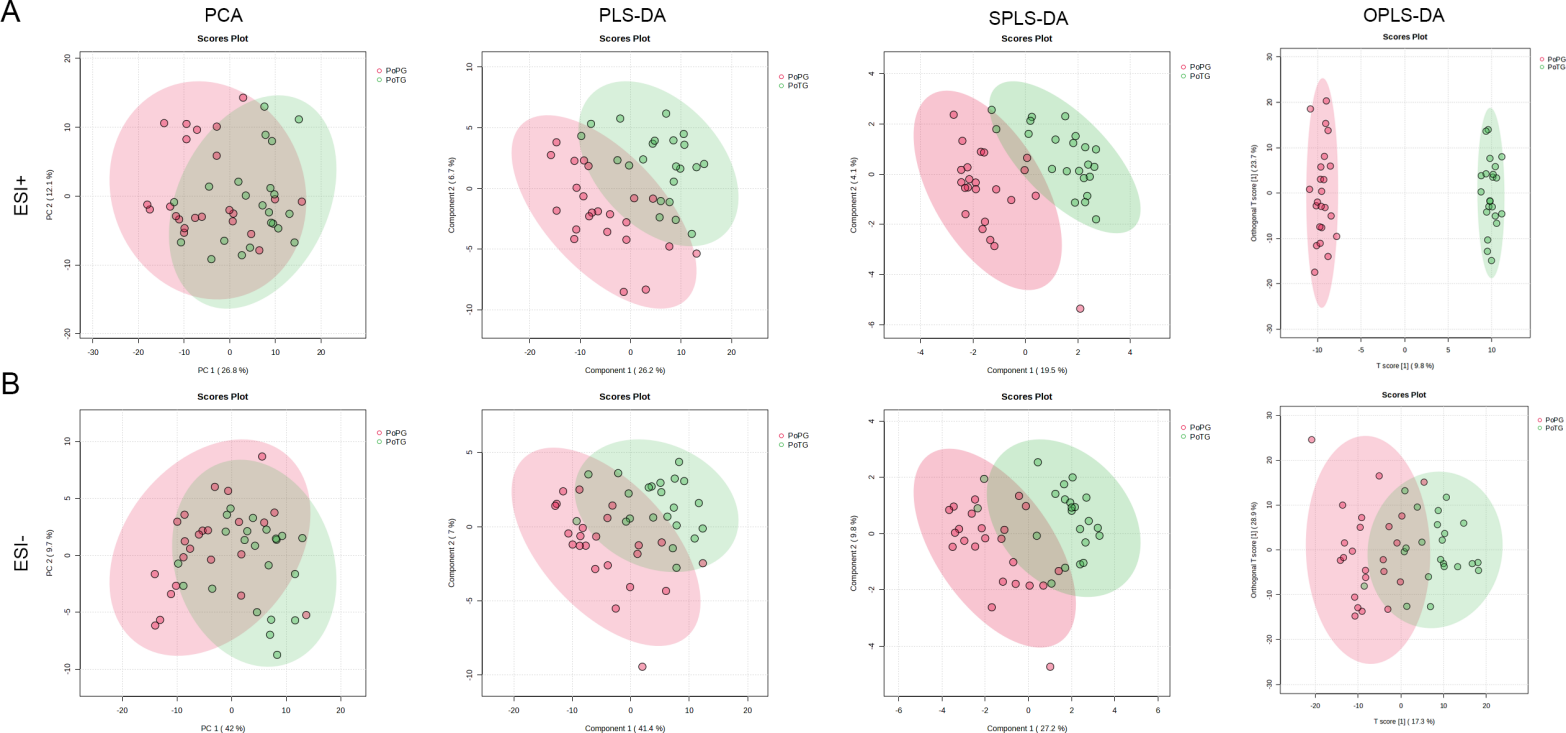
The alteration in vaginal metabolites in pregnant women with preterm birth after cervical cerclage. **(A)** PCA, PLS-DA, SPLS-DA, and OPLS-DA in ESI+ modes. **(B)** PCA, PLS-DA, SPLS-DA, and OPLS-DA in ESI− modes.

Untargeted metabolomics based on LC-Orbitrap-MS/MS was carried out to elaborate the differences in metabolites between pregnant women with PTB and term birth. As shown in **Figure 5A**, the first and second components (PC1 and PC2) of the PCA score plot (*R* ^2^ = 0.294, and *p* = 0.001) accounted for 26.8% and 12.1% in ESI+ mode, respectively. Meanwhile, PCA exhibited distinct clustering of the vaginal microbiota in pregnant women with PTB, suggesting that there were remarkable differences in vaginal metabolites between pregnant women with PTB and term birth. Subsequently, the results of PCA were further confirmed by PLS-DA (*R* ^2^ = 0.382 and *Q* ^2^ = 0.243), SPLS-DA (Classification error rate = 0.178), and OPLS-DA (*R* ^2^ *Y* = 0.995, and *Q* ^2^ = 0.416). In ESI− mode, PC1 and PC2 of PCA score plots (*R* ^2^ = 0.223 and *p* = 0.001) accounted for 42.0% and 9.7%, respectively (**Figure 5B**). The results of PCA displayed that the distribution points of samples in the PTB and TB groups were relatively clustered, and there were distinct discrepancies between the two groups. PLS-DA (*R* ^2^ = 0.338 and *Q* ^2^ = 0.239), SPLS-DA (Classification error rate = 0.244), and OPLS-DA (*R* ^2^ *Y* = 0.626, and *Q* ^2^ = 0.339) were applied to further analyze the alteration in vaginal metabolites. The two groups could be divided into two distinct regions (PTB and TB groups), confirming that the vaginal metabolism of the PTB group was significantly different from that of the TB groups.

### 3.6 Screening for the differential metabolic features

**Figure 6:**
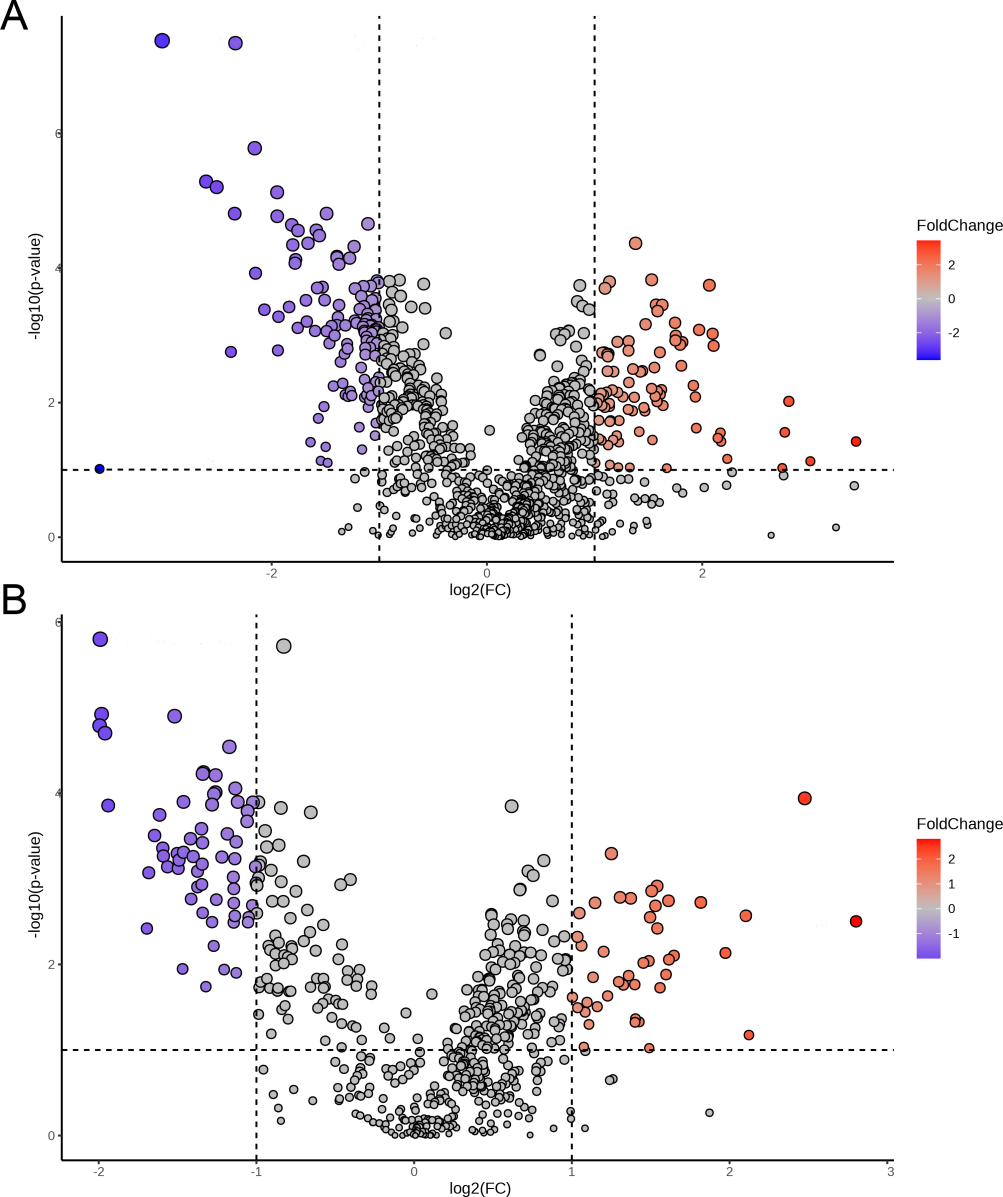
The volcano plot for vaginal metabolites between the TB and PTB groups in the ESI+ **(A)** and ESI− **(B)** modes.

Volcano plots were used to analyze the differential metabolic features between the PTB and TB groups based on univariate statistical analysis with criteria of VIP > 1, *p* < 0.05, and fold change (FC) ≥2. As shown in **Figure 6A** and **Supplementary Figure S2**, a total of 204 differential metabolic features were selected and identified in ESI+ mode, including 114 differential metabolic features that were reduced in the PTB group compared with the TB group, and 90 differential metabolic features were increased. Meanwhile, a total of 112 differential metabolic features were screened and identified in ESI− mode, of which 67 differential metabolic features were reduced in the PTB group compared with the TB group, and 45 differential metabolic features were increased (**Figure 6B** and **Supplementary Figure S3**).

**Figure 7:**
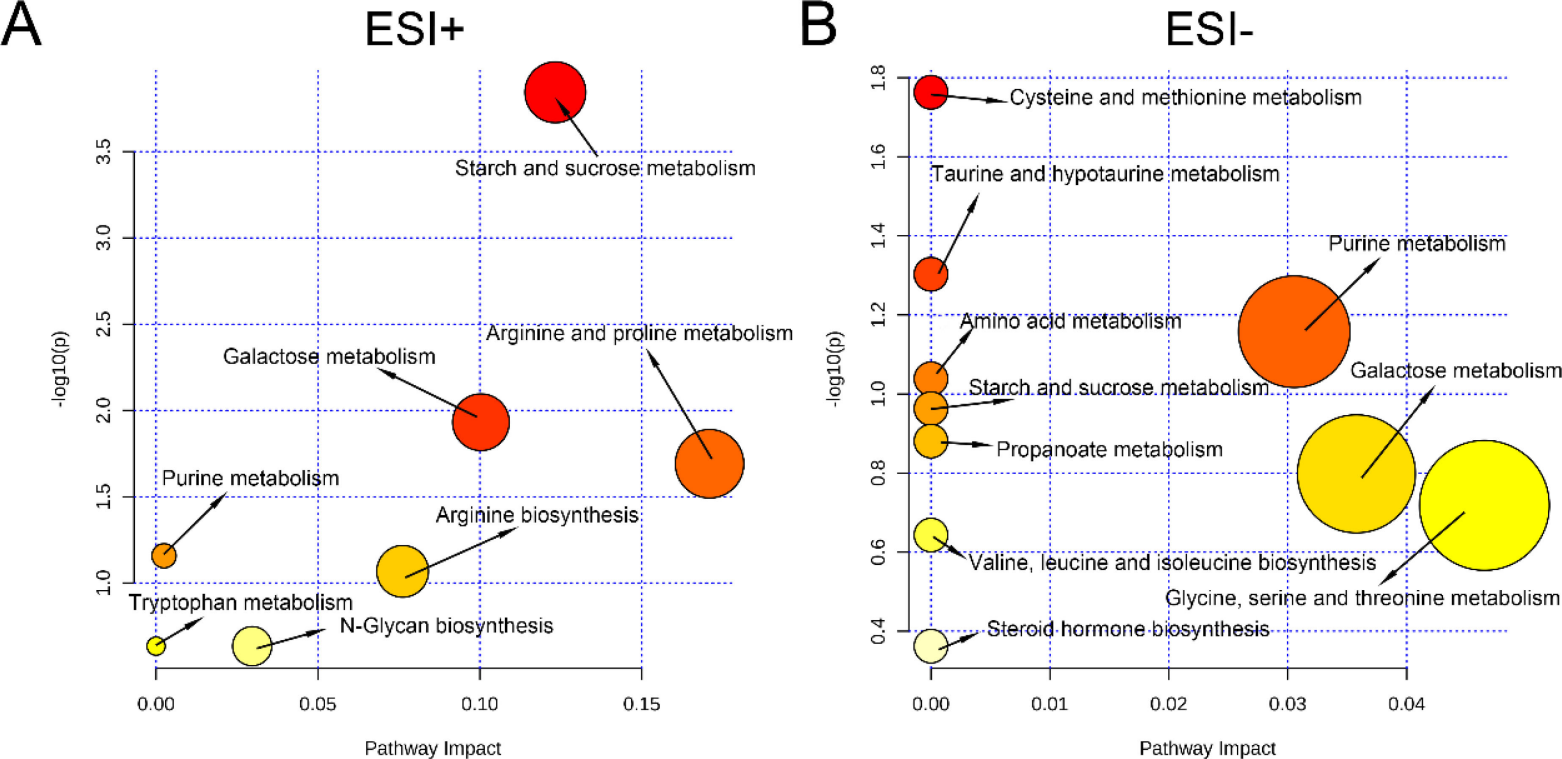
Analysis of key metabolic pathways. **(A)** Pathway analysis in the ESI+ modes. **(B)** Pathway analysis in the ESI− modes.

To investigate which metabolic pathways were involved in the alteration of vaginal metabolic features, pathway analysis was conducted based on the KEGG database. In ESI+ mode, seven potential key metabolic pathways associated with PTB were revealed, namely, starch and sucrose metabolism, arginine and praline metabolism, galactose metabolism, purine metabolism, arginine metabolism, tryptophan metabolism, and N-glycan biosynthesis (**Figure 7A**). In addition, 10 potential key metabolic pathways associated with PTB in ESI− mode, namely, cysteine and methionine metabolism; taurine and hypotaurine metabolism; purine metabolism; amino acid metabolism; galactose metabolism; starch and sucrose metabolism; propanoate metabolism; valine, leucine, and isoleucine biosynthesis; glycine, serine, and threonine metabolism; and steroid hormone biosynthesis (**Figure 7B**).

## 4 Discussion

According to a previous report, about three-quarters of the cases are diagnosed as spontaneous PTBs, which include previous spontaneous PTB or preterm prelabor rupture of the membranes (Humberg et al., 2020). Without an effective approach of identifying pregnancies at risk for PTB, it remains difficult to target interventions or clinical trials. Recently, vaginal microbiota have been confirmed to be associated with the risk of PTB in single-center studies, accelerating the idea of utilizing the vaginal microbiome to establish reliable predictive models to identify pregnancies at risk for PTB. Therefore, this study aimed to explore the relationship between key differential microbiota/metabolites and PTB.

Some studies indicated that the high diversity of the gut microflora is beneficial for improving the host health, but it is widely accepted that the low diversity of the vaginal microflora is positively related to women’s health (Anahtar et al., 2018). Although the vaginal microbiota composition is strongly associated with race, geographic background, health status, and pregnant women, *Lactobacillus* (*L. iners*, *L. crispatus*, *L. gasseri*, or *L. jensenii*) is the major microorganism in the vagina due to the remarkable elevation in circulating estrogen (Ravel et al., 2011). In reproductive-age women, the proportion of *Lactobacillus* is significantly higher than that in others. *Lactobacillus* given in beneficial amounts as a live biotherapeutic is a beneficial bacterium for the host’s microbiota as shown by the administration of *L. crispatus* to prevent recurrent bacterial vaginosis (Cohen et al., 2020). *Lactobacillus* processes are proven to have a series of physiological effects, such as suppressing oxidative stress, regulating intestinal microbiota, and inhibiting inflammatory responses (Qin et al., 2024). *Lactobacillus* also prevents the growth of harmful bacteria in the vagina by elevating the levels of short-chain fatty acids. In addition, [*Ruminococcus*] *gnavus group*, *Megamonas*, *Catenibacterium*, and *Holdemanella* act as short-chain fatty acid (SCFA)-producing bacteria, which are beneficial for host health (Liu et al., 2021; Shimizu et al., 2023). *Roseburia* supplementation suppressed inflammatory responses and restored the intestinal barrier by inhibiting the activation of the NLRP3 inflammasome and upregulating the expression of tight junctional protein, respectively (Seo et al., 2020). Conversely, *Desulfovibrionaceae* is regarded as one of the most important members among lipopolysaccharide producers, as it can destroy the integrity of the intestinal barrier and induce inflammatory responses (Liu et al., 2024). *Eubacterium* has been reported to promote the secretion of inflammatory cytokines and suppress the activity of antioxidant enzymes in mice with nonalcoholic fatty liver disease (Zhuge et al., 2022). *Gardnerella* is an anaerobic and Gram-variable pleomorphic bacillus that is found in women of childbearing age. However, a high proportion of *Gardnerella* cause a series of diseases, including PTB, fetal growth restriction, and invasive neonatal infections (Wong et al., 2022). The alteration in vaginal microbiota may be associated with the occurrence of PTB.

Untargeted metabolomics could offer a comprehensive view of the metabolic alterations between pregnant women with PTB and term birth. Ranolazine is an active piperazine derivative that promotes the β-oxidation of fatty acid and transforms into the oxidation of carbohydrates, thereby regulating inflammatory responses and suppressing oxidative stress (Redondo-Muñoz et al., 2023). Miglitol is the traditional anti-diabetic drug that regulates bile acid metabolism, which is beneficial for preventing the occurrence of PTB (Hamada et al., 2013; Shah et al., 2023). Celastrol, Fumigaclavine A, and Pimecrolimus inhibit proinflammatory cytokine secretion and oxidative stress (Li et al., 2023; Zhang et al., 2021; Du et al., 2011; Kocanci et al., 2024). Doxapram is a respiratory stimulatory analeptic drug that has been applied for decreasing apnea and hypoxic episodes in preterm infants (Flint et al., 2021). Hydroxyphenyllactic acid is a tyrosine metabolite that stems from *L. plantarum*, which suppresses the growth of harmful bacteria (Mu et al., 2010). Conversely, N1,N8-diacetylspermidine is a minor component of human urinary polyamines and is widely used as a tumor marker for breast and colorectal cancers (Umemori et al., 2010). High contents of N-acetylputrescine can cause gut microbiota disorders and the occurrence of inflammatory responses, and it elevates the risk of some diseases, including sepsis and Parkinson (Fulcher et al., 2022). Inosine is an endogenous purine nucleoside that suppresses inflammatory responses and maintains oxidative homeostasis (Guo et al., 2021; Preitner et al., 2013). Melibiose is composed of galactose and glucose, and reported to increase melibiose levels in rats with acute cholestasis (Tomsik et al., 2008). Isoetharine is extensively applied for improving asthma and chronic asthmatic bronchitis, while high levels of isoetharine promote an increase in heart rate (Maciag et al., 2022). 3-Hydroxyisovaleric acid stemmed from the degradation of leucine, which causes the occurrence of mitochondrial toxicity by destroying the balance of esterified CoA and free CoA (Brown et al., 2016). In addition, the administration of *L. crispatus* may decrease some inflammatory markers (Armstrong et al., 2022) and shift the vaginal microbiota toward an optimal state (Cohen et al., 2020). These results indicate that alterations in vaginal metabolites are strongly associated with the occurrence of PTB.

## 5 Conclusion

In summary, the influences of vaginal microbiota and metabolites on the outcome of pregnant women after cervical cerclage were revealed using metabolomic analysis. High-throughput sequencing revealed that the proportion of beneficial bacterium was reduced and the proportion of harmful bacterium increased in the PTB group compared with the TB group. According to the results of untargeted metabolomics, 316 differential metabolic features between the PTB and TB group were identified as potential biomarkers in both ESI+ and ESI− modes, and these differential metabolic features were enriched in 14 metabolic pathways. The present study provides distinct vaginal microbiome and metabolome profiles in women with preterm delivery following cervical cerclage, which is beneficial for establishing a predictive model for PTB.

## Data Availability

The original contributions presented in the study are publicly available. This data can be found here: NCBI, PRJNA1122359.
