# Supplementary material for: Distinct vaginal microbiome and metabolome profiles in women with preterm delivery following cervical cerclage
Source: Front Cell Infect Microbiol. 2025 Feb 11;15:1444028. doi: 10.3389/fcimb.2025.1444028 (PMC11850995; doi:10.3389/fcimb.2025.1444028)

**Supplementary data**

**Figure S1** Robust principal component analysis (RPCA) of beta-diversity values.


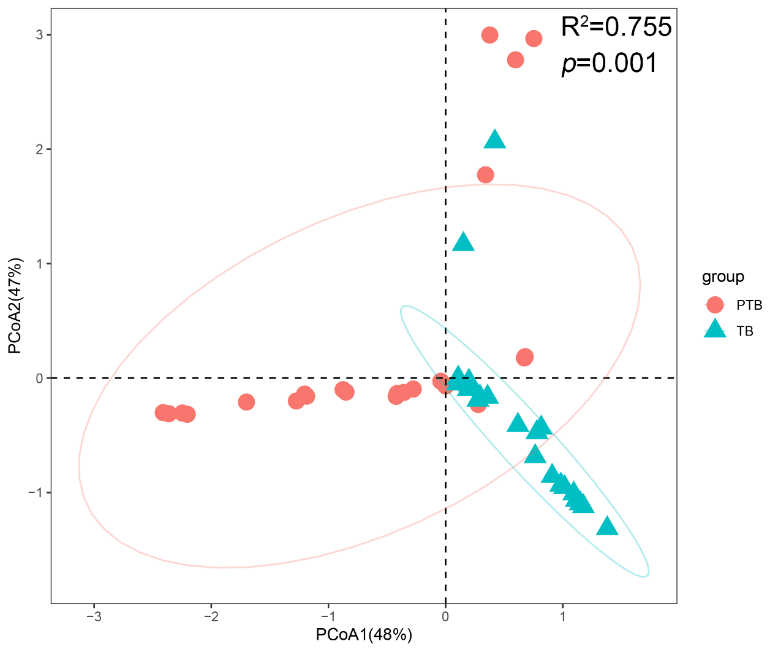


**Figure S2** Heatmap of 204 differential metabolic features between the PTB and TB group in the ESI+ modes. The blue presented the low value, white presented the mid value, and red presented the high value.


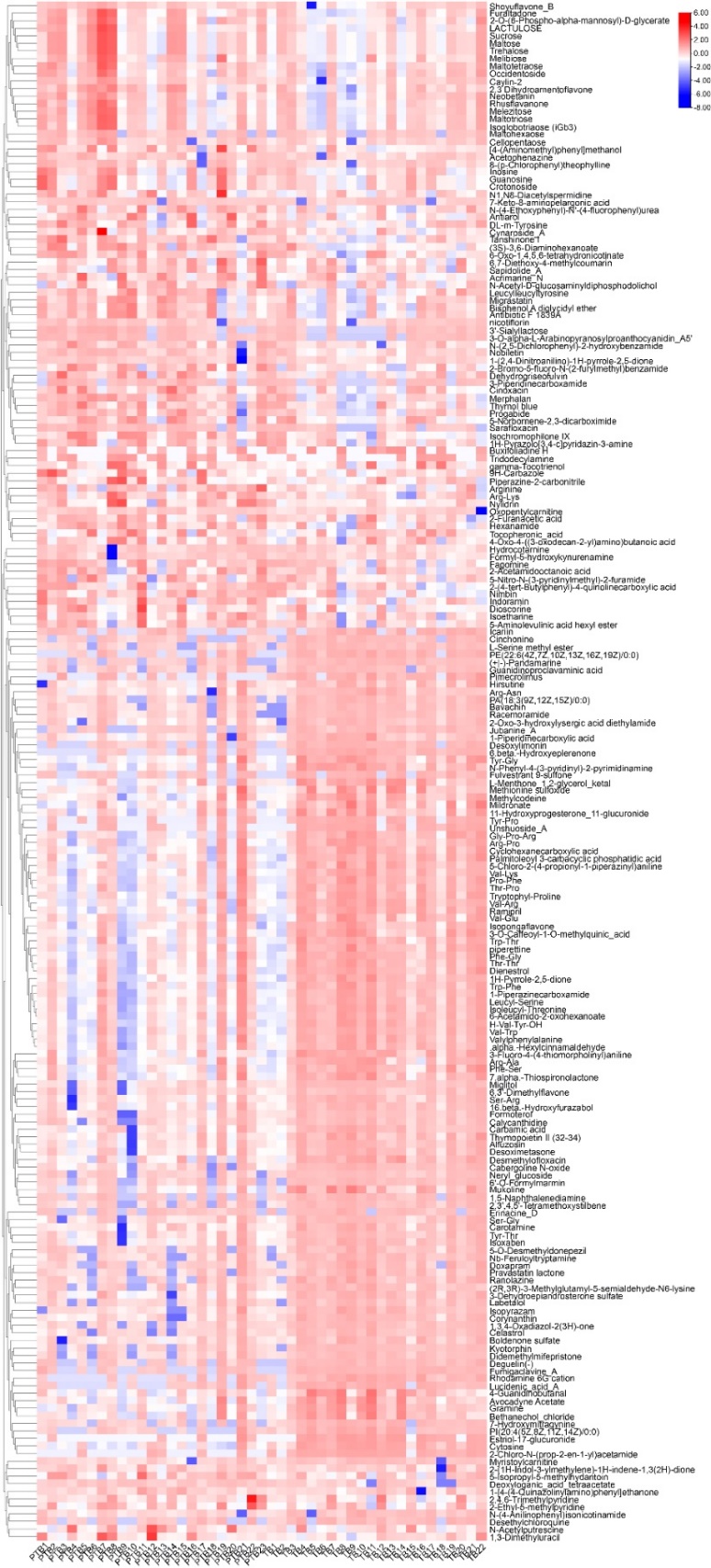


**Figure S3** Heatmap of 112 differential metabolic features between the PTB and TB group in the ESI- modes. The blue presented the low value, white presented the mid value, and red presented the high value.


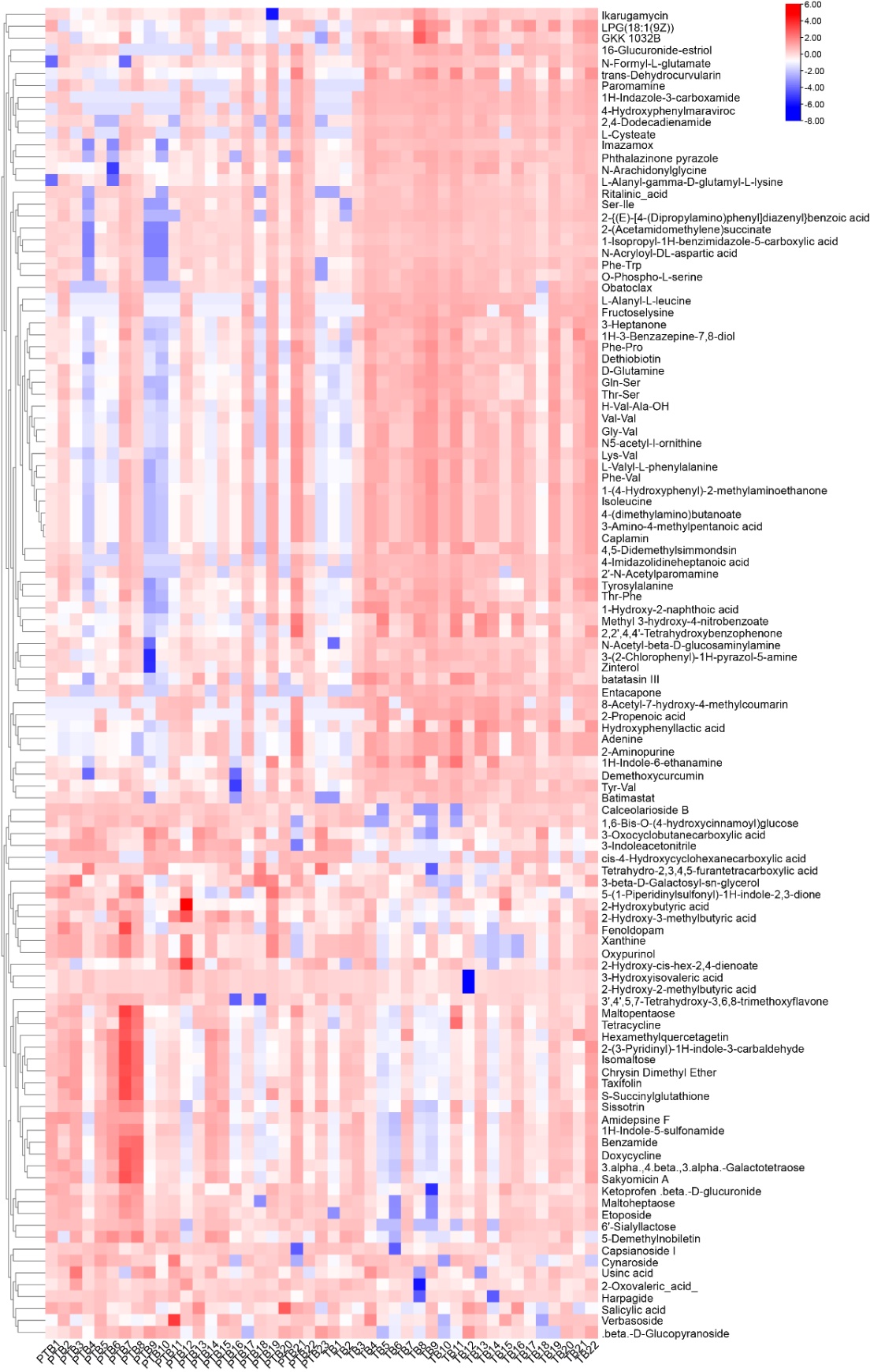

Supplement: Supplementary file 1 [file Table1.docx]
